# A mathematical model for targeting chemicals to tissues by exploiting complex degradation

**DOI:** 10.1186/1745-6150-6-46

**Published:** 2011-09-22

**Authors:** Bruce S Gardiner, Lihai Zhang, David W Smith, Peter Pivonka, Alan J Grodzinsky

**Affiliations:** 1 School of Computer Science and Software Engineering, The University of Western Australia, WA, 6009, Australia; 2 Department of Infrastructure Engineering, University of Melbourne, VIC 3010, Australia; 3 Center for Biomedical Engineering, Department of Electrical Engineering and Computer Science, and Department of Mechanical Engineering, Massachusetts Institute of Technology, Cambridge, Massachusetts, USA

**Keywords:** Solute transport, Tissue, Mathematical model, Prodrug, Insulin-like Growth factor (IGF), proteases

## Abstract

**Background:**

In many biological and therapeutic contexts, it is highly desirable to target a chemical specifically to a particular tissue where it exerts its biological effect. In this paper, we present a simple, generic, mathematical model that elucidates a general method for targeting a chemical to particular tissues. The model consists of coupled reaction-diffusion equations to describe the evolution within the tissue of the concentrations of three chemical species: *a *(concentration of free chemical), *b *(binding protein) and their complex, *c *(chemical bound to binding protein). We assume that all species are free to diffuse, and that *a *and *b *undergo a reversible reaction to form *c*. In addition, the complex, *c*, can be broken down by a process (*e.g. *an enzyme in the tissue) that results in the release of the chemical, *a*, which is then free to exert its biological action.

**Results:**

For simplicity, we consider a one-dimensional geometry. In the special case where the rate of complex formation is small (compared to the diffusion timescale of the species within the tissue) the system can be solved analytically. This analytic solution allows us to show how the concentration of free chemical, *a*, in the tissue can be increased over the concentration of free chemical at the tissue boundary. We show that, under certain conditions, the maximum concentration of *a *can occur at the centre of the tissue, and give an upper bound on this maximum level. Numerical simulations are then used to determine how the behaviour of the system changes when the assumption of negligible complex formation rate is relaxed.

**Conclusions:**

We have shown, using our mathematical model, how complex degradation can potentially be exploited to target a chemical to a particular tissue, and how the level of the active chemical depends on factors such as the diffusion coefficients and degradation/production rates of each species. The biological significance of these results in terms of potential applications in cartilage tissue engineering and chemotherapy is discussed. In particular, we believe these results may be of use in determining the most promising prodrug candidates.

## Background

A fundamental problem in biology and medicine is how to target a chemical to the particular tissue where it is to exert its biological action. Examples of chemicals to be targeted include endogenous hormones, growth factors and prescribed drugs. The advantage of targeting a drug to a particular tissue is that potential side-effects of the undesirable presence of the drug in other tissues are minimised.

The results of a previous theoretical study describing the transport of IGF suggested a means of achieving selective targeting to particular tissues. Selective degradation of the IGF-IGFBP complex was shown to be capable of regulating the free concentration of IGF (the biologically active form) within a tissue. In some cases the free IGF concentration in the tissue was raised to a level an order of magnitude or more greater than in the adjacent synovial fluid [1]. We believe that adjusting the rate of degradation of the complex may well provide a generic mechanism for tuning tissue exposure to various chemicals in the body.

In order to gain further insight into these processes, in this paper we perform a parametric study on a generic system consisting of two molecules and their complex diffusing within a tissue. We use a combination of mathematical analysis and numerical simulations to conduct 'mathematical experiments' to investigate the effect of the diffusion coefficients, degradation rates, binding affinities, *etc*. on the uptake of molecules. Consistent with the previous study of [1], the transport of IGF, IGFBP and their small complex in cartilage are considered. We also apply our model to the transport of a prodrug within a tumour, which provides another important illustration of this effect.

The primary source of circulating IGF-1 is the liver and its serum concentration is relatively constant throughout the day in healthy adults [2,3]. The interactions between IGFs and several high-affinity IGF-binding proteins (IGFBPs) are important for IGF transport [4-6] from the liver to its site of biological action. In solution, binding proteins (IGF-BPs) act as 'carrier proteins', forming IGF-IGFBP complexes, which prolong the half-life of IGFs [7]. For example, in serum the majority of IGF-1 is bound to IGFBP3 [8]. Our recent theoretical study investigated the role that diffusible binding partners may play in regulating transport in a tissue [1]. Compared to the absence of a binding partner, it was shown that the existence of a complex which is broken down within the tissue, releasing free IGF, may lead to a substantial increase in the free IGF concentration within the tissue, even to concentrations well above those at the tissue boundary. Such a mechanism is biologically plausible as IGFBP-degrading proteases, such as matrix metalloproteases (MMPs), are capable of cleaving IGFBP into fragments that have a low binding affinity for IGFs, releasing functional free IGF from the complex [5,9].

However, there are numerous other examples where this mechanism for targeting a chemical to a particular tissue can be exploited, particularly in drug delivery *e.g. *of chemotherapeutic agents to solid tumours. To be effective, the drug must be able to reach the tumour in sufficient concentrations to kill the malignant cells, but it is desirable to minimise exposure of healthy cells to the drug, as it may cause tissue damage or lead to other adverse side effects. Some treatments may be targeted by injection directly into the tumour, but most current types of chemotherapy are administered intravenously, or orally, and so must reach their target via the circulatory system [10]. This route exposes healthy and malignant cells alike to the drug. The problem is exacerbated by the fact that solid tumours frequently contain poorly vascularised regions, which means the drug must travel further from the blood vessel to reach its site of biological action. Further, advective transport with the interstitial fluid may be reduced as a consequence of leaky vessels within the tumour raising interstitial fluid pressure [10]. These factors, together with the fact that the drug is consumed within the tissue, means that those areas furthest from the blood vessel generally receive the lowest dose.

To overcome these difficulties, a variety of approaches are being pursued to increase the concentration of the drug within the tumour, including magnetic targeting (which appears best suited to tumours near the surface of the body) [11,12], or the use of macrophages attracted to hypoxic regions as vehicles for drug delivery [13]. However, some chemotherapeutic agents take the form of a prodrug, which is administered in an inactive form and subsequently metabolised to an active form in the body. Experimental prodrugs (including tirapazamine (TPZ), AQ4N and PR-104) have been developed which are reduced to a cyctotoxic form only under hypoxic conditions, preventing a build-up of the active drug in well-oxygenated cells [10,14]. In terms of our model, we can view the prodrug as being a complex, which is broken down into an active, cyctotoxic form, and a by-product.

Existing mathematical models for the transport and metabolism of the prodrug TPZ suggest that it does not penetrate efficiently into the tumour tissue [15,16], which appears puzzling in the light of some promising pre-clinical and clinical results [10] (although some negative results have also been reported recently [17]). However, these past models do not contain equations to describe the distribution of the active drug. Here, we apply our model to the transport of a generic prodrug within a tumour. Our analysis shows that the concentration profiles of the prodrug and its active form may be dramatically different, and hence it is important to look at both when considering the likely effectiveness of the drug. This might at least partly to explain the apparent paradox with regard to TPZ penetration. More generally, an improved theoretical understanding of the various factors that affect the ability of the prodrug to target the tumour would allow for more rapid identification of the characteristics of the most promising drug candidates.

This paper is organised as follows: in Methods, we present the model equations, which comprise a coupled system of three reaction-diffusion equations for the concentrations of two chemicals and their complex. Model predictions are presented in Results and Discussion. We first consider the special case in which the rate of formation of the complex within the tissue is small. Under this assumption, we are able to solve the model analytically, which allows us to show explicitly how the concentrations of each species depend upon the various parameters. In this case, we are able to show that, under certain conditions on the parameters, the maximum concentration of active chemical (IGF or active drug) can occur in the centre of the tissue, and give an upper bound for this concentration. We then present numerical simulations of the full model, showing how increasing the complex formation rate affects the solutions. The paper concludes with a summary of our main results, and suggested directions for future research.

## Methods

**Figure 1:**
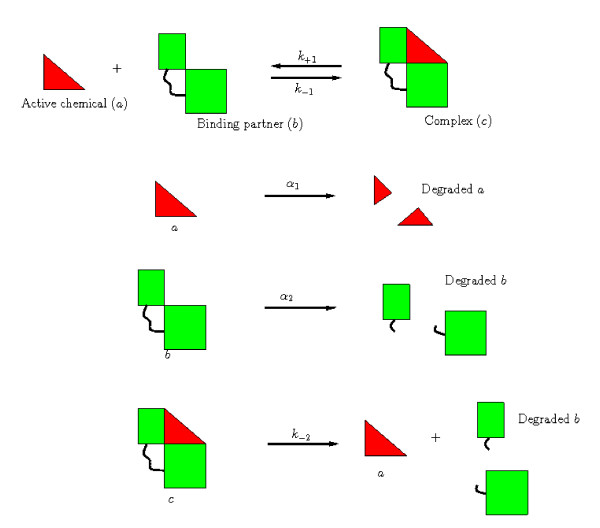
**Diagrammatic representation of the reactions (1)**.

We develop a mathematical model of a simple generic system in which three chemicals, *a*, *b *and *c*, react and diffuse within a region of tissue, Ω. We assume *a *and *b *can undergo a reversible reaction to form a complex, *c*, the rates of association and dissociation being *k*_+1 _and *k*_-1_. Both *a *and *b *are assumed to be removed from the tissue at constant rates, *α*_1 _and *α*_2_, respectively. Note that, throughout this paper, we use the term 'removal' to describe all processes which result in a particular species being unable to participate in further interactions; such processes can include degradation, consumption or conversion to an inactive form. The complex may also be broken down by a reaction which results in the release of *a*, without a corresponding release of *b *(*e.g. *an enzymatic reaction which degrades *b *within the complex). This reaction occurs at a rate *k*_-2_. These reactions are summarised diagrammatically in Figure 1.

We denote the concentrations of the three chemicals, *a*, *b *and *c *at a position ***x ***∈ Ω by *A *(***x***, *t*), *B *(***x***, *t*) and *C *(***x***, *t*) (where *t *is time). Each chemical diffuses within the tissue, with diffusion coefficients *D_A_*, *D_B _*and *D_C _*respectively. Then, applying the law of mass action, we obtain the following system of reaction-diffusion equations for the chemical concentrations

(1a) $$\frac{\partial A}{\partial t}=D_{A}\nabla^{2}A-k_{+1}AB+\left(k_{-1}+k_{-2}\right)C-\alpha_{1}A,$$

(1b) $$\frac{\partial B}{\partial t}=D_{B}\nabla^{2}B-k_{+1}AB-\alpha_{2}B+{k_{-}}_{1}C,$$

(1c) $$\frac{\partial C}{\partial t}=D_{C}\nabla^{2}C+k_{+1}AB-\left(k_{-1}+k_{-2}\right)C.$$

Initially, we assume the chemical concentrations are given by

(2) $$A\left(x,0\right)=A_{i}\left(x\right),\;B\left(x,0\right)=B_{i}\left(x\right),\;C\left(x,0\right)=C_{i}\left(x\right),$$

whilst on the tissue boundary we set

(3) $$A=A^{*},\;B=B^{*},\;C=C^{*}\;\text{on}\;\partial \Omega .$$

In terms of the cartilage example, we view *a *as representing IGF, *b *as IGF binding protein 3 (IGFBP3), and *c *as the small, IGF-IGFBP3 binary complex. Our model thus represents a simplified version of models previously presented in [1,18]. In terms of the prodrug example, we take *c *to be the prodrug, whilst *a *represents the active form of the drug, with its removal being interpreted as consumption by the tumour cells. In this example, *b *does not play an active role in the chemotherapy, and represents a by-product of the reduction of the produg.

### Nondimensionalisation

We nondimensionalise equations (1) as follows (with tildes indicating dimensionless quantities)

$$x=L\tilde{x},\;t=\frac{L^{2}\tilde{t}}{D_{C}},\;A=A^{*}\tilde{A},\;B=B^{*}\tilde{B},\;C=C^{*}\tilde{C}.$$

where *L *is typical lengthscale of the tissue, and A*, B* and C* are the concentrations of *a*, *b *and *c*, respectively, at the tissue boundary. The timescale chosen is the timescale for the complex, *c*, to diffuse through the domain. The dimensionless equations are then (dropping tildes)

(4a) $$\frac{\partial A}{\partial t}=\delta_{A}\nabla^{2}A-\lambda_{1}\mu_{A}AB+\lambda_{2}\mu_{A}C-\lambda_{3}A$$

(4b) $$\frac{\partial B}{\partial t}=\delta_{B}\nabla^{2}B-\lambda_{1}\mu_{B}AB-\lambda_{4}B+\lambda_{5}C$$

(4c) $$\frac{\partial C}{\partial t}=\nabla^{2}C+\lambda_{1}AB-\lambda_{2}C$$

where we have introduced the dimensionless parameters

$$\begin{array}{c} \lambda_{1}=\frac{L^{2}k_{+1}A^{*}B^{*}}{C^{*}D_{C}},\;\lambda_{2}=\frac{L^{2}\left(k_{-1}+k_{-2}\right)}{D_{C}},\\ \lambda_{3}=\frac{\alpha_{1}L^{2}}{D_{C}},\;\lambda_{4}=\frac{\alpha_{2}L^{2}}{D_{C}},\;\lambda_{5}=\frac{L^{2}{k_{-}}_{1}C^{*}}{B^{*}D_{C}},\\ \delta_{A}=\frac{D_{A}}{D_{C}},\;\delta_{B}=\frac{D_{B}}{D_{C}},\;\mu_{A}=\frac{C^{*}}{A^{*}},\;\mu_{B}=\frac{C^{*}}{B^{*}}. \end{array}$$

The *λ_i _*(*i *= 1, 2, ..., 5) represent the ratios of the timescale of complex diffusion to timescales of the various reactions: *λ*_1 _is the relative rate of complex formation; *λ*_2 _and *λ*_5 _are the relative rates of complex breakdown to yield *a *and *b*, and *λ*_3 _and *λ*_4 _are the dimensionless rates of removal of *a *and *b*. The ratio of the diffusivities of *a *and *b *compared to that of *c *are given by *δ_A _*and *δ_B _*respectively, and *μ_A _*and *μ_B _*are the ratios of the concentration of complex to the concentration of *a *and *b *at the boundary.

The solutions of the model equations are presented in the following section. In certain cases the model can be simplified sufficiently to be solved in closed form. This provides both validation of, and complementary insight to, the numerical solutions of the full model.

## Results and Discussion

For simplicity, we consider a one-dimensional geometry, where the tissue is taken to occupy the region -1 ≤ *x *≤ 1 and we assume the problem to be symmetric about *x *= 0. This geometry was assumed in previous studies of IGF transport in cartilage [1,18], and hence facillitates comparison with earlier results. In terms of the prodrug example, we could take it as being representative of a microenvironment inside a tumour where there are blood vessels located at *x *= -1 and *x *= 1 (see *e.g. *[19], where a similar geometry was adopted). Note that our assumption of symmetry about *x *= 0 allows us to restrict our attention to 0 ≤ *x *≤ 1, with the boundary conditions (3) modified to

(5a) $$\frac{\partial A}{\partial x}=\frac{\partial B}{\partial x}=\frac{\partial C}{\partial x}=0\;\text{at}\;x=0,$$

(5b) A=B=C=1atx=1.

We restrict our attention to the steady-state solutions of the governing equations, since we are interested in the long-term behaviour of the system. For the purposes of the numerical simulations, we set

(6) A(x, 0) = B(x,0) = C(x,0) = 1,

which is the simplest choice consistent with the boundary conditions (5).

### Analysis for small complex formation rate (λ_1 _≪ 1)

We now make the further assumption that the dimensionless rate of complex formation (*λ*_1_) is small. For the case of IGF, this appears plausible, since taking the parameter estimates from [1] (A* ~ 10^-11 ^M, C* ~ 10^-9 ^M, *L *~ 10^-1 ^cm and *k*_+1 _~ 10^5 ^M^-1 ^s^-1^) and assuming B* to be of a similar order of magnitude to A*, and *D_C _*to be of the order of magnitude of *D_A _*(*D_A _*~ 10^-7 ^cm^2 ^s^-1^, again from [1]) gives *λ*_1 _~ 10^-3^. For prodrugs, we likewise believe the assumption to be plausible (though it is not possible to give a quantitative estimate based on data from the literature). Typically, the prodrug must be converted to a radical to be active, and under well oxygenated conditions, the unpaired electron on the radical is rapidly transferred to molecular oxygen, preventing build-up of the active drug. In the hypoxic environment of the tumour, however, this transfer cannot occur, so the rate at which the prodrug is regenerated is much lower [14]. Hence we expect the prodrug concentration will be much higher than those of its breakdown products in the blood (so A* B*/C* will be small), whilst the rate of prodrug re-formation in the hypoxic environment of the tumour will be slow (hence *L*^2 ^*k*_+1_/*D_C _*will also be small). Throughout this section, we thus take *λ*_1 _≪ 1 in (4) (with all other parameters assumed *O*(1)). This simplification allows us to solve the governing equations analytically, which provides a clear insight into how complex degradation affects the distribution of the active chemical in the tissue. The effect of relaxing this assumption is considered in the following section.

For subsequent convenience, we write the solutions as the sum of steady state and time dependent parts, so that

$$\begin{array}{c} A=A_{T}\left(x,t\right)+A_{S}\left(x\right),\;B=B_{T}\left(x,t\right)+B_{S}\left(x\right),\\ C=C_{T}\left(x,t\right)+C_{S}\left(x\right). \end{array}$$

Since any difference between the initial and steady state chemical distributions will decay exponentially fast, we confine our attention to the steady state solutions here. We expand the steady state components as a power series in the small parameter *λ*_1 _so that

(7) $$A_{S}=A_{0}\left(x\right)+\lambda_{1}A_{1}\left(x\right)+\ldots ,$$

and similarly for *B_S _*and *C_S _*.

Substituting the above into the governing equations (4) we find the leading-order solutions obey

(8a) $$\delta_{A}\frac{\partial^{2}A_{0}}{\partial x^{2}}+\lambda_{2}\mu_{A}C_{0}-\lambda_{3}A_{0}=0,$$

(8b) $$\delta_{B}\frac{\partial^{2}B_{0}}{\partial x^{2}}-\lambda_{4}B_{0}+\lambda_{5}C_{0}=0,$$

(8c) $$\frac{\partial^{2}C_{0}}{\partial x^{2}}-\lambda_{2}C_{0}=0.$$

(We note that the leading-order time-dependent solutions can also be obtained analytically by straightforward separation of variables and application of Sturm-Liouville theory (see *e.g. *[20]). The solutions of (8) are given by

(9a) $$A_{0}=\left\{ \begin{array}{l} \left(1+\frac{\mu_{A}\lambda_{2}}{\delta_{A}\lambda_{2}-\lambda_{3}}\right)\frac{\cosh(\sqrt{\frac{\lambda_{3}}{\delta_{A}}}x)}{\cosh(\sqrt{\frac{\lambda_{3}}{\delta_{A}}})}\\ -\frac{\mu_{A}\lambda_{2}}{\delta_{A}\lambda_{2}-\lambda_{3}}\frac{\cosh(\sqrt{\lambda_{2}x})}{\cosh(\sqrt{\lambda_{2}})})\;\text{if}\;\delta_{A}\lambda_{2}\neq \lambda_{3},\\ \left(1+\frac{\mu_{A}\sqrt{\lambda_{2}}\tanh(\sqrt{\lambda_{2}})}{2\delta_{A}}\right)\frac{\cosh(\sqrt{\lambda_{2}}x)}{\cosh(\sqrt{\lambda_{2}})}\\ -\frac{\mu_{A}\sqrt{\lambda_{2}}}{2\delta_{A}}\frac{x\;\sinh(\sqrt{\lambda_{2}}x)}{\cosh(\sqrt{\lambda_{2}})}\;\text{if}\;\delta_{A}\lambda_{2}=\lambda_{3}, \end{array}\right.$$

(9b) B0=1+λ5(δBλ2-λ4)coshλ4δBxcoshλ4δB-λ5 cosh(λ2x)(δBλ2-λ4)cosh(λ2)ifδBλ2≠λ4,1+λ52δBλ2 tanhλ2coshλ2xcoshλ2-λ52δBλ2xsinhλ2xcoshλ2ifδBλ2=λ4,

(9c) $$C_{0}=\frac{\cosh\left(\sqrt{\lambda_{2}}x\right)}{\cosh\left(\sqrt{\lambda_{2}}\right)}.$$

We begin by considering the case *λ*_1 _= 0, for which the steady solutions are given exactly by (9), in order to investigate the effects of the other parameters. Since the equations for *A*_0 _and *B*_0 _are similar in form, we focus on the behaviour of the solutions for *A*_0 _and *C*_0_. We begin by noting the following basic points. Firstly, in the absence of reactions (*λ_i _*= 0, *i *= 1, 2, ... 5), *A*_0 _= *B*_0 _= *C*_0 _= 1 - *i.e*. the steady state solution is spatially uniform and the concentrations are equal to their values at the boundary. When there is no breakdown of the complex to produce *a *and *b *(*i.e*. *λ*_2 _= *λ*_5 _= 0), *A*_0 _and *B*_0 _have a minimum at *x *= 0, since they are depleted in the tissue due to removal. Provided *λ*_2 _> 0, *C*_0 _has a minimum at *x *= 0, since it is only supplied at the boundary, but breaks down throughout the tissue.

**Figure 2:**
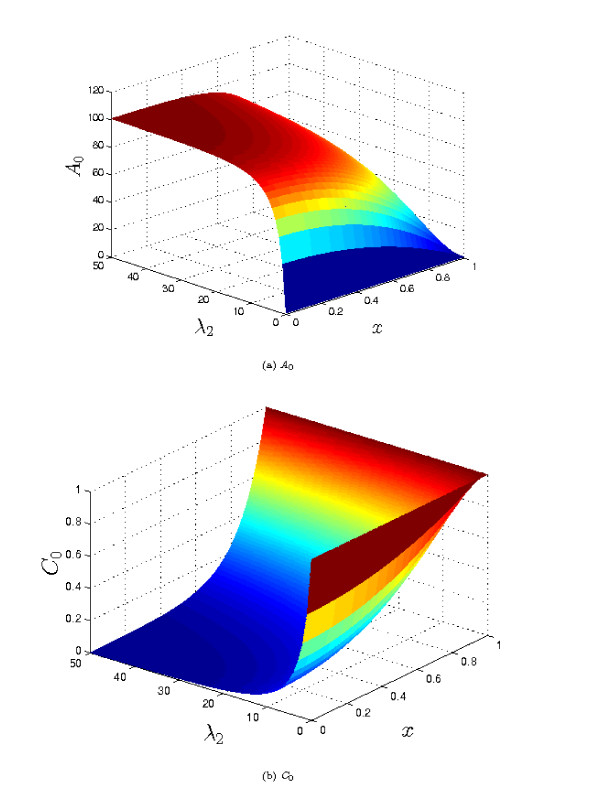
**Plots of the steady state solutions *A*_0 _and *C*_0 _for varying values of *λ*_2 _(see equation (9))**. Other parameter values: *λ*_3 _= 0, *μ_A _*= 100, *δ_A _*= 1. Although the concentration of the complex, *c*, declines rapidly away from the tissue boundary, the level of a is rapidly increasing. Thus, for the prodrug example, apparently poor prodrug penetration into the tissue can, in fact, correspond to high levels of active drug in the tissue, due to efficient conversion of the prodrug to active form.

If the aim is to maximise the concentration of *a *throughout the tissue, intuition suggests that we need to reduce *λ*_3_/*δ_A _*to as small a value as possible (*i.e*. minimise the rate of removal relative to the rate of diffusion of *a*). Taking *λ*_3 _= 0, it is then clear from equation (9a) that the largest values of *A*_0 _will occur when *λ*_2 _is large, (*i.e*. when the rate of complex breakdown is fast compared to its rate of diffusion). Furthermore, the maximum value of *A_S _*occurs at *x *= 0, in the centre of the tissue. In the limit *λ*_2 _→ ∞, the maximum value of *A*_0 _→ 1+ *μ_A_*/*δ_A_*, and is attained throughout most of the tissue (except for within a thin boundary layer, of width $O(\lambda_{2}^{-\frac{1}{2}}))$ close to *x *= 1, where the concentration of *a *falls to unity). Correspondingly, *C*_0 _→ 0 in most of the tissue, except in the thin layer at the tissue's edge. This type of behaviour is illustrated in Figure 2. We note that, in this case, the level of *a *in the tissue does not depend upon the rate of complex breakdown (which is rapid), but is limited only by the supply of *c *to the tissue (which occurs by diffusion), and 'escape' of *a *at the edge of the tissue. Hence increasing the supply of *c*, either by increasing its concentration at the boundary, or by increasing its diffusivity results in increased levels of *a *within the tissue, as does reducing the diffusivity of a (which reduces the amount lost at the boundary). From the point of view of prodrug delivery to tumours, looking only at the concentration profile for *c *might lead us to think that the penetration of the prodrug is very poor (*C*_0 _is only nonzero in a thin layer close to the boundary). However, when the concentration plot of the active drug (*A*_0_) is considered, we would expect the prodrug treatment to be highly effective, as the active drug concentration is high throughout most of the tumour.

**Figure 3:**
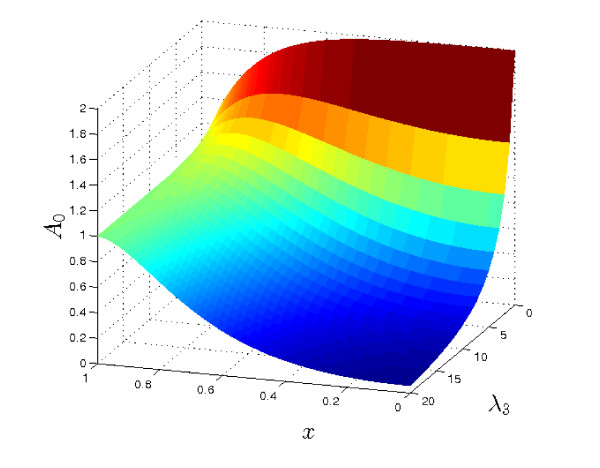
**Plots of the steady state solutions *A*_0 _for various values of *λ*_3 _(see equation (9a))**. Other parameter values: *λ*_2 _= 50, *μ_A _*= *δ_A _*= 1. For small *λ*_3 _the centre of the tissue (*x *= 0) has the highest concentration of *a*; as *λ*_3 _increases, the maximum point moves towards the tissue's edge (*x *= 1), until for sufficiently large *λ*_3_, the concentration is monotonically decreasing away from the boundary.

For *O*(1) values of *λ*_3 _(*i.e*. where the rate of consumption or removal of *a *is appreciable), we can observe three general types of behaviour. For the purposes of illustration, we set *λ*_2 _= 50, *λ*_4 _= *λ*_5 _= 0, *μ_A _*= *μ_B _*= *δ_A _*= *δ_B _*= 1 (recall that *λ*_1 _= 0 throughout this section), and vary *λ*_3_. The results are plotted in Figure 3. For relatively small values of *λ*_3_, *A_S _*has a single maximum, in the centre of the tissue. (Note it is straightforward to show that if *x *= 0 is a maximum of *A*_0_, then *A*_0 _has no other fixed points.) Conversely, for large values of *λ*_3_, *A*_0 _has a single minimum at the centre of the tissue. At intermediate values, we see the third type of behaviour: *A*_0 _has two extrema - a minimum at *x *= 0 and a maximum somewhere else in the tissue.

**Figure 4:**
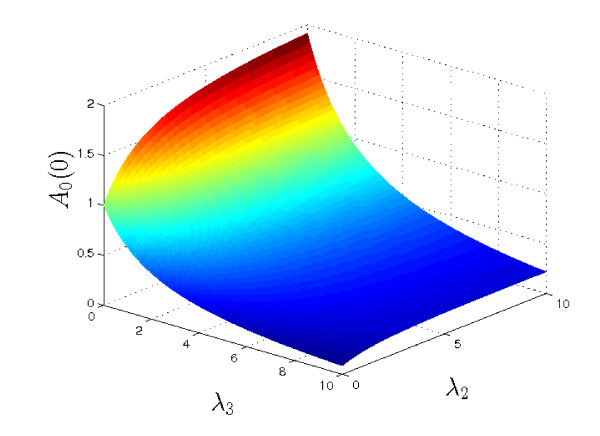
**Plot showing *A*_0_(0) (see equation (9a)) for a range of values of *λ*_2 _and *λ*_3 _(with *μ_A_*/*δ_A _*= 1)**. Note that the largest values of *A*_0_(0) are achieved when *λ*_3 _= 0 (no removal/consumption of *a*) and *λ*_2 _is large (rapid breakdown of *c*), in which case *A_S _*(0) → 1 + *μ_A_*/*δ_A _*= 2. Hence the highest levels of *a *at the centre of the tissue occur when the rate of complex breakdown (*λ*_2_) is rapid, and the rate of consumption of *a *(*λ*_3_) is minimised.

In terms of prodrug treatment for tumours, the aim would be to achieve the greatest possible level of active drug in the centre of the tumour (*i.e*. to maximise *A*_0_(0)). Figure 4 shows a plot of *A*_0_(0) for a range of values of *λ*_2 _and *λ*_3_. As might be expected, the maximum concentration of *a *occurs when there is no consumption/removal (*λ*_3 _= 0) and the rate of breakdown of the complex, *c*, is large. In this case we notice *A*_0_(0) → 1 + *μ_A_*/*δ_A _*= 2 - *i.e*. supply of *c *becomes the limiting factor.

We now consider how the behaviour illustrated above changes when *λ*_1 _≠ 0. For small values of *λ*_1 _(assuming all other parameters are *O *(1)), we can determine the effect of complex formation on the distributions of the three chemicals by considering the next-order correction terms *A*_1_, *B*_1 _and *C*_1 _in the expansion (7). The general solutions are

(10a) A1=A*coshλ3δAx+ ∑j=16Aj coshθjx(1)+A7 cosh(2λ2x)+A8 cosh(λ2x),(2)(3)

(10b) B1=B*coshλ4δBx+ ∑j=16Bj coshθjx+B7 cosh(2λ2x)+B8+B9 cosh(λ2x),

(10c) C1=C*coshλ2x+ ∑j=16Cj cosh(θjx)+C7 cosh(2λ2x)+C8,

where the *θ_i _*are given by:

(11a) $$\theta_{1}=\sqrt{\frac{\lambda_{3}}{\delta_{A}}}+\sqrt{\frac{\lambda_{4}}{\delta_{B}}},\;\theta_{2}=\sqrt{\frac{\lambda_{3}}{\delta_{A}}}-\sqrt{\frac{\lambda_{4}}{\delta_{B}}},$$

(11b) $$\theta_{3}=\sqrt{\frac{\lambda_{3}}{\delta_{A}}}+\sqrt{\lambda_{2}},\;\theta_{4}=\sqrt{\frac{\lambda_{3}}{\delta_{A}}}-\sqrt{\lambda_{2}},$$

(11c) $$\theta_{5}=\sqrt{\frac{\lambda_{4}}{\delta_{B}}}+\sqrt{\lambda_{2}},\;\theta_{6}=\sqrt{\frac{\lambda_{3}}{\delta_{B}}}-\sqrt{\lambda_{2}}.$$

For the sake of concision, the full details of the solutions, including coefficients $A_{j}$, $B_{j}$, $C_{j}$*etc*. are given in the Appendix.

**Figure 5:**
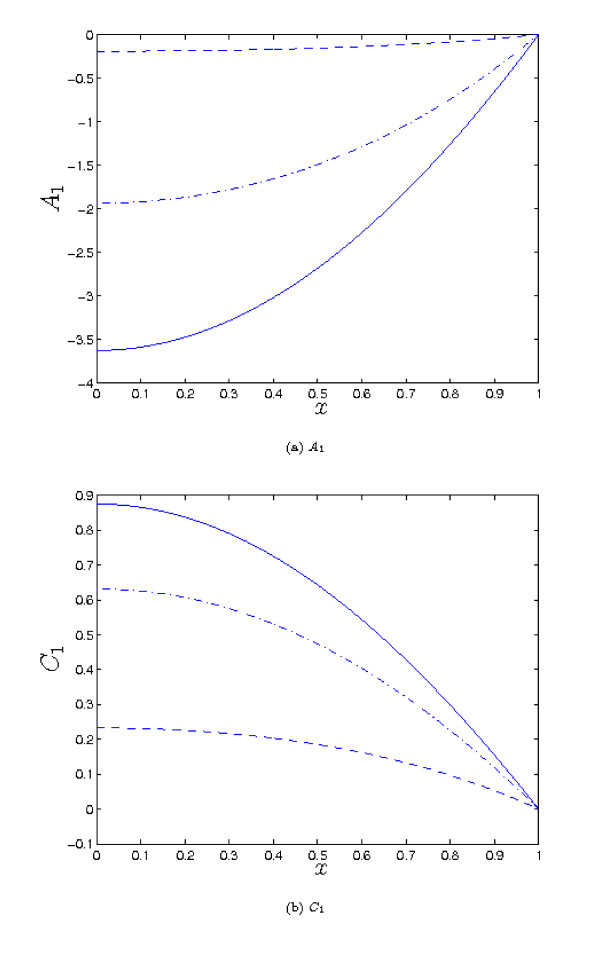
**Plots showing the first-order correction terms *A*_1 _and *C*_1 _for various parameter values (see equation (10))**. Dashed line: *λ*_2 _= 5, *λ*_3 _= 0.5, *λ*_4 _= 0.2, *μ_A _*= 1. Dot-dashed line: *λ*_2 _= 2, *λ*_3 _= 1.5, *λ*_4 _= 0.2, *μ_A _*= 1. Solid line: *λ*_2 _= 1, *λ*_3 _= 0.5, *λ*_4 _= 0.2, *μ_A _*= 5. In all cases we take: *λ*_4 _= 0.2, *λ*_5 _= *mu_B _*= *δ_A _*= *δ_B _*= 1

Plotting the above correction terms for a range of parameter values, as illustrated in Figure 5, we observe that in general the effect of small but non-zero *λ*_1 _is exactly what would be anticipated: a reduction in the concentration of *a *throughout the tissue, with a corresponding increase in the concentration of *c*. The effect is greatest in the centre of the tissue.

### Numerical simulations of the full model

For the sake of completeness, we now turn to the general case where the parameter values are unrestricted, which requires the model equations (4) to be solved numerically, subject to the boundary and initial conditions (5)-(6). We use a semi-implicit finite difference method in Matlab. The solutions were verified by checking the results for small values of *λ*_1 _against the analytical results obtained in the previous section, and were found to give excellent agreement (data not shown). The simulations presented in this section use a timestep *dt *= 0.005 and a spatial step *dx *= 0.002, and were run until the solution reached steady state.

**Figure 6:**
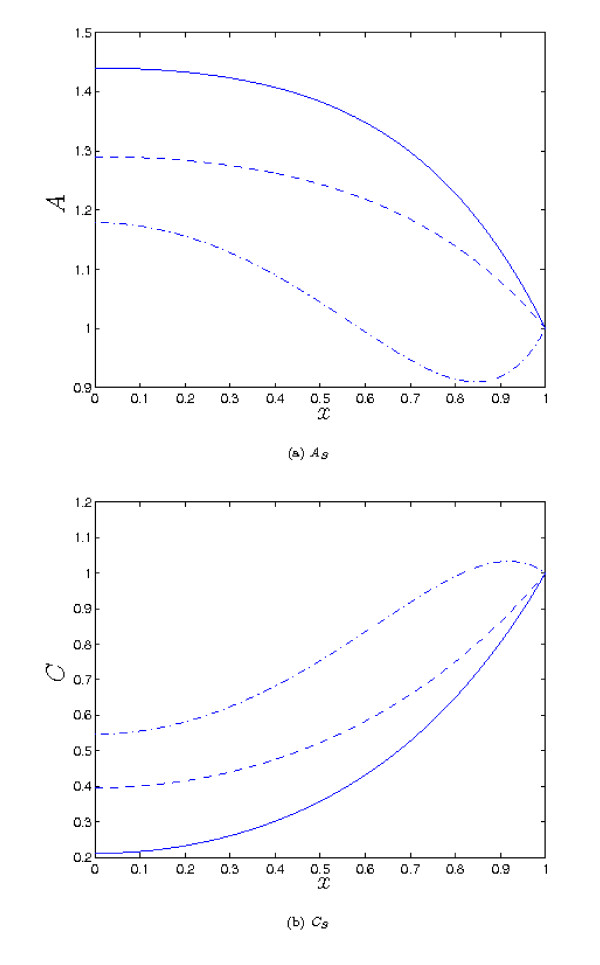
**Plots of the steady state solutions for *A *and *C***. Parameter values: *λ*_1 _= 0 (solid), *λ*_1 _= 2 (dash), *λ*_1 _= 20 (dot-dash). Other parameter values: *λ*_2 _= 5, *λ*_3 _= 0.5 *λ*_4 _= *λ*_5 _= *μ_A _*= *μ_B _*= *δ_A _*= *δ_B _*= 1 in all cases. Increasing the rate of complex formation (*λ*_1_) leads to a reduction in *A*, and corresponding increase in *C*. We also note *A *can now have a maximum at *x *= 0 and another extremum in 0 <*x *< 1, in contrast to the *λ*_1 _= 0 case.

**Figure 7:**
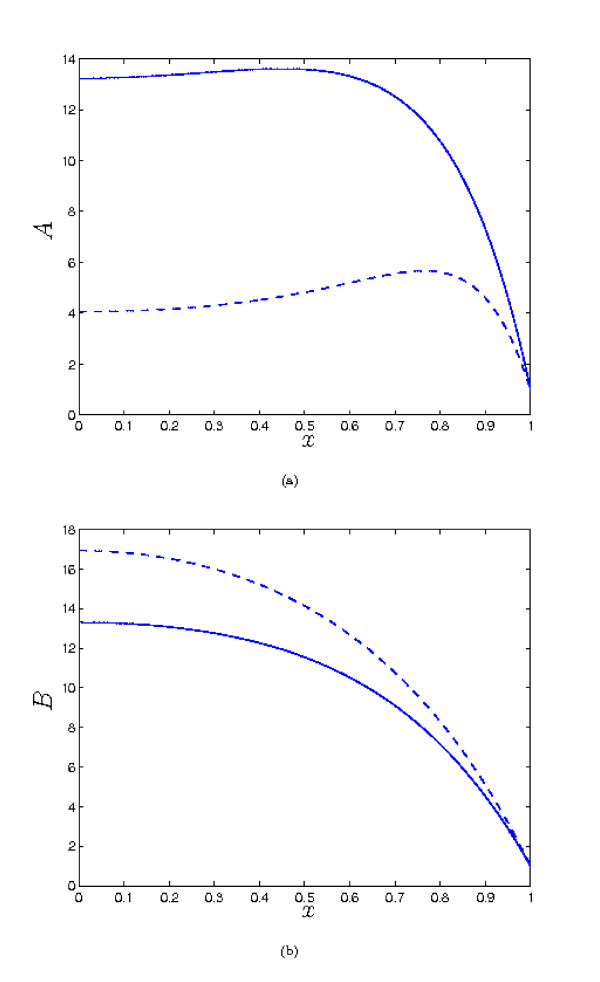
**Plots of the steady state solution for *A *and *B***. Parameter values: *λ*_1 _= 0 (solid), *λ*_1 _= 0.01 (dash). Other parameter values: *λ*_2 _= 1 *λ*_3 _= *λ*_4 _= 0.5, *λ*_5 _= 12, *μ_A _*= 10, *μ_B _*= 0.2, *δ_A _*= 0.02, *δ_B _*= 0.1. In this case, since *A *and *B *are relatively large (*B *= *O*(10^1^) in both cases; data for not shown), small values of *λ*_1 _can make a significant difference to the concentration profile.

**Figure 8:**
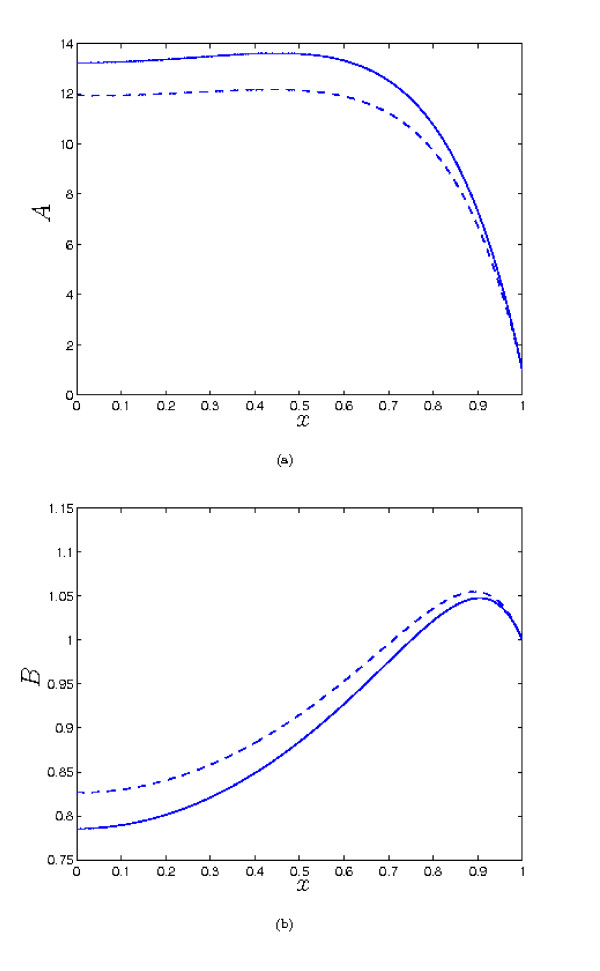
**Plots of the steady state solutions for *A *and *B***. Parameter values: *λ*_1 _= 0 (solid), *λ*_1 _= 0.01 (dash). Other parameter values: *λ*_2 _= 1, *λ*_3 _= 0.5 *λ*_4 _= 10, *λ*_5 _= 12, *μ_A _*= 10, *μ_B _*= 0.2, *δ_A _*= 0.02, *δ_B _*= 0.1. Compared to Fig. 7, we observe that decreasing the level of *b *(through increasing *λ*_4_) increases the concentration of *a*, since the rate of complex formation is reduced.

As we saw in the previous section, increasing *λ*_1 _reduces the the concentration of *a *within the tissue, and correspondingly increases the level of *c *compared to the *λ*_1 _= 0 case. However, when the parameter values are *O *(1), moderate increases in *λ*_1 _do not make a great difference to the qualitative behaviour of the solutions, as we see from from Figure 6. It is necessary to increase *λ*_1 _by an order of magnitude to observe such a change - *e.g. *as shown in Figure 6 for λ_1 _= 20, it is now possible for *A *to have a second fixed point when *x *= 0 is a maximum, in contrast to what was found in the previous section, for λ_1 _= 0. When the other parameters are not *O *(1), *A *and *B *can be large, and small values of λ_1 _can make a significant difference to the concentrations e.g. see Figure 7 (note that in this case, our small λ_1 _analysis is not valid, as that requires the other parameters to be *O *(1)). But here it is important to note that the complex formation term introduces significant coupling between the solutions for *A *and *B*, so the binding partner now plays a role in determining the concentration of *a*, rather than simply being a passive product of complex breakdown. For example, repeating the simulation of Figure 7, but with increased λ_4 _(the dimensionless rate of removal of *b*) results in a decreased level of *b *and hence an increased concentration of *a *(compare Figures 7 and 8). This is because there is now less *b *available to 'tie up' *a *by forming the complex. Hence, we would suggest that to achieve a high concentration of *a*, it is generally desirable that the binding partner should be removed as rapidly as possible from the tissue.

## Conclusions

In this study, we have considered a simple mathematical model for the interplay of diffusion, degradation and complex formation and breakdown in the transport of two chemical factors and their complex. Interactions between these processes can produce behaviours very different to what is possible when complex breakdown does not occur. Theoretically it is clear that the key to targeting one of these chemicals (*a*) to a particular tissue (and specifically, to the centre of that tissue) is to ensure the complex breakdown rate is large (whilst the formation rate is small), and the removal rate of *a *is as small as possible. In addition the boundary concentration and diffusivity of the complex relative to those of *a *should also be as large as possible, and the removal rate of the binding partner *b *should be large, so it does not persist in the tissue and 'tie up' *a *by forming the complex. In certain parameter regimes, it is not only possible to raise the concentration of a in the centre of the tissue to a level much higher than that outside, but also for the concentration of *a *to be maximised there. This may be significant in terms of the treatment of hypoxic regions of tumours (see below). More generally, varying the rate of complex breakdown and the rate of removal can give different concentration profiles for *a *in the tissue. This might also conceivably be used to provide positional information to regulate cell behaviour *e.g. *during development.

The results presented here complement an earlier modelling study which considered the transport of IGF, IGFBP and their complex in cartilage [1]. We have shown that, generally speaking, fast breakdown of the complex enhances the level of IGF in the tissue. However, when the rate of breakdown becomes very fast, the concentration of IGF is limited by the balance of complex entering the tissue, and IGF leaving the tissue, embodied in the parameter ratio *μ_A_*/*δ_A _*in our model. It is suggested that tissues may exploit the processes considered here to 'tune' their exposure to various factors. As our results show, this tuning could be effected by adjusting the rate of complex breakdown, or the rate of removal of *a *(*e.g. *through regulation of enzyme activity or receptor concentration). (It appears less likely that the tissue would be able to influence the diffusivities or boundary concentrations of the chemicals, though changes in extracellular tissue matrix density and composition may exert some effect.) Importantly, this mechanism can give relatively uniform levels of *a *through most of the tissue (as seen in Figure 2), which may be necessary for maintaining tissue homeostasis (*e*.*g*. for the prescence of a hormone or growth factor in a tissue).

In the context of the development of prodrugs for treating tumours, our results suggest that not only might it be possible to increase the active drug concentration in the tumour to levels far above those in the circulation, the centre of the tumour, generally the most hypoxic region, can be preferentially targeted. Compared to some earlier models (*e.g. *[15,16]), which consist only of a reaction-diffusion equation for the concentration of prodrug, our model can provide new insights when applied to prodrug transport. Our results show that the distribution of the active drug may be very different from that of the prodrug, and that considering only the latter can be misleading. We have seen that low levels of prodrug in the centre of a tumour do not necessarily imply poor penetration. They may, in fact, be a very positive sign, associated with high levels of active drug in that region, due to complete conversion of the prodrug to active form. This may explain the promising pre-clinical and clinical results of the prodrug TPZ referred to in [10], despite previous modelling suggesting it does not penetrate efficiently into the tumour [15,16]. However, the rate of consumption of the active drug has an important role in determining its concentration profile: low rates are required for a high concentration of the active drug at the tumour centre. Higher rates of drug consumption drag the drug concentration profile down. Indeed, very high consumption rates result in the situation that occurs for conventional therapies, where the active drug concentration declines rapidly with distance from the blood vessel. At intermediate consumption rates, the drug concentration in the tumour is greatest at some point between the boundary and the centre of the tumour using this model of drug behaviour. Nevertheless, high consumption rates, say by receptor-ligand binding so dragging down drug concentration profiles, implies that the drug is actually doing what it is suppose to. When combined with experiments to determine the diffusion coefficients and reaction rates of the various species (along the lines of those presented in [15,16]), the theoretical results presented here may be of assistance in determining which potential new prodrugs are most likely to be effective, by excluding those *e.g. *for which the diffusivity is too low, the rate of reduction to active form too slow, or the rate of consumption of the active drug too great. Whilst more complex scenarios for prodrug activation can be envisaged, we believe a strength of our generic model is that it gives a useful insight into the fundamental principles involved, whilst being simple enough to be understood on an intuitive level. It can also provide a foundation for the development of more detailed models to elucidate more complicated situations as needed. More speculatively, we believe our results suggest another potential avenue for drug delivery. Where a naturally occurring chemical and binding partner (interacting in the way assumed in this paper) can be found in a tissue of interest, a drug might be designed in such a way that it forms a stable complex with the binding partner. The drug could then 'piggy-back' on this native binding protein, to be released preferentially in the tissue of interest.

## Competing interests

The authors declare that they have no competing interests.

## Authors' contributions

BSG, LZ, DWS and PP developed the mathematical model and were involved in the interpretation of results and drafting of the paper; AJG contributed biological background to the development of the mathematical model.

All authors read and approved the final manuscript.

## Reviewers' comments

### Reviewer's Report 1

Dr Artem Novozhilov (nominated by Eugene V. Koonin)

#### Main Comments

In this manuscript the authors present a mathematical model that simulate interaction and diffusion of three chemicals. It is shown by means of analytical and numerical investigation that there are such parameter values that, given the initial and boundary conditions, it is possible to observe the maximum concentration of one of the chemical in the middle of the spatial domain, which is supposed to represent a tissue. The modeling results are discussed in terms of potential applications in cartilage tissue engineering and chemotherapy. In this review I would like to refrain from discussing possible implications of the modeling results with respect to, e.g., chemotherapy, and focus my critique on the mathematical analysis of the system of three reaction-diffusion equations formulated in the manuscript. In general, I would recommend the authors to present their mathematical results in a more coherent and exact form.

Here are my comments listed in no particular order.

1. The authors first state that they consider the boundary conditions of the form A = A*, B = B*, C = C* (Eq. (3) on page 3). On page 4 they state that "... our assumption of symmetry about *x *= 0 allows us to restrict our attention to 0 ≤ *x *≤ 1 with the boundary condition (3) modified to $\frac{\partial A}{\partial x}=\frac{\partial B}{\partial x}=\frac{\partial C}{\partial x}=0$ at *x *= 0 at *x *= 1" These are two different mathematical problems. These problems can be proved to be equivalent only in the case of identical initial conditions for *x *< 0 and *x *> 0, which is generally not supposed in the paper, or in the case of identical asymptotical behavior of solutions for *x *< 0 and *x *> 0, which is not proved in the manuscript. Therefore the jump from the first problem to the second one requires some discussion.

Response: *We agree the wording of this section was not sufficiently precise. We have re-worded this to: '... and we assume the problem to be symmetric about x = *0.' *which we hope makes our intention clear*.

2. The authors should elaborate on why they suppose that at the ends of the spatial domain all three chemicals have fixed concentrations. It can be supposed that one of the chemicals is targeted to the tissue; can we assume the same about the other two chemicals?

Response: *The assumption of constant levels of A, B and C on the tissue boundary, although a simplification is, we believe, reasonable for the following reasons. Firstly, in the case of IGF (and some other hormones), its levels in the blood remain fairly constant throughout the day, as mentioned in the introduction. The binding partner is also fairly constant throughout the day and so the complex concentrations can also be reasonably approximated as constant. In the case of a drug treatment, we believe our assumption is a reasonable approximation for the levels in the blood when the prodrug is administered intravenously over a relatively long period (e.g. hours). It would also be reasonable for in vitro experiments where the tissue is placed in a large stirred bath of solution containing the treatment. Of course, in reality there will be examples of growth factors and drug administration regimes where temporal variation in the concentrations at the boundary is important. However, we wished to consider a simple, generic example here; such effects could be studied in future work*.

3. On page 4 the authors write (I copy the sentence verbatim) "We restrict our attention to the steadystate solutions of the governing equations, since we are interested in the long-term behaviour of the system, and any different between the initial condition and the steady state will decay [. . . ]" The long-term behavior of a dynamical system is characterized by the type of attractor it tends to; the attractors can be steady states (both spatially homogeneous and non-homogeneous), cycles (periodic solutions) or chaotic. I don't fully understand why it is a priory possible to restrict the attention to only one type of long-term behavior.

Response: *We agree that in general several long-term behaviours are possible in dynamical systems, with steady states being just one example. However, physical intuition would suggest in this case the system would settle to a steady state, and in an applied paper of this nature, we do not feel a great deal would be gained by establishing this by rigorous mathematical proof. We note our intuition is borne out by numerical simulations*.

4. The next quote from the text (page 4): "As a result, the initial conditions are immaterial". If there are, for instance, two asymptotically stable steady states then the initial conditions become important.

Response: *We agree with the reviewer that when there are two asymptotically stable steady states, initial conditions are not immaterial, and we have revised the text accordingly. From numerical simulations, however, we have not been able to find more than one asymptotically stable steady state in this case*.

5. The authors state (Abstract) that "In the special case where the rate of complex formation is small [. . . ] the system can be solved analytically." Actually, the authors solve the system analytically only in the case when the rate of complex formation is zero. For this parameter value the system is linear, and therefore the steady-state solution can be written down in an explicit analytical form. Even in this case, however, it is necessary to prove that this steady state solution is stable to study the longterm behavior of the system (numerical experiments show that it is stable).

Response: *We agree with the reviewer notes that, in general, when studying the long-time behaviour of dynamical systems it is important to identify which (if any) steady states are stable. However, considering the multidisciplinary audience of this journal, we believe too much technical mathematical content may be off-putting to many readers. In the case λ_1 _= *0, *the steady-state equations are linear and so their solution is unique. Of course, this does not prove that the system will evolve to this steady state, but given the simple processes involved, physical intuition would lead us to expect this, and it is borne out by the numerical simulations. We thus believe our approach is reasonable, although lacking mathematical rigour. We would argue similarly in the nonlinear (λ_1 _>*0*) case*.

6. In the case when the rate of complex formation is zero (*λ*_1 _= 0) the authors show, using the explicit solution, that it is possible to have the maximum of concentration at the left boundary. This conclusion is hardly surprising because, in words, the following system is considered: There is a constant inflow of the chemical from the right boundary, this chemical diffuses and degrades, and there is an impenetrable boundary at the left side. If the chemical does not degrade with a high rate it is clear that the concentration at the left boundary will increase. This conclusion does not need any explicit math. Therefore, it would be interesting to see which other insights can be obtained with the help of the suggested system of differential equations.

Response: *The reviewer suggests that a verbal argument suffices to understand the fact that, when λ*_1 _= 0, the active drug concentration can have a maximum at the centre of the tissue. We agree, but would point out that not all the results of the model, even in this simpler case, may not be so clear on an intuitive level. For example, the fact that the maximum drug concentration may occur at a point intermediate between the centre and the edge of the tissue. If such an observation was made experimentally, we do not believe it would necessarily be intuitively obvious that it could be produced by the simple mechanism outlined here. We also see there is a maximum theoretically attainable active drug concentration (A* + D_C _C*/D_A _in dimensional terms), which again might not be intuitively clear, and obviously has practical implications.

**Figure 9:**
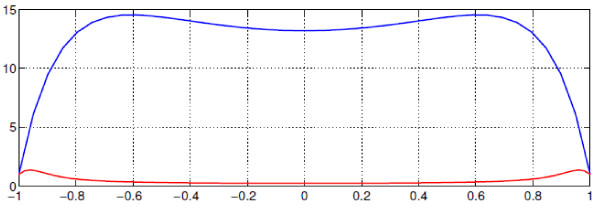
**Numerical simulation provided by Reviewer 1**.

7. The authors argue that the found analytical solution is very close, in case of small *λ*_1_, to the steadystate solution observed in the numerical experiments. The agreement, according to the text, is excellent, but they do not show the data. Let me consider the following values of the parameters: *λ*_1 _= 0.1, *λ*_2 _= 1, *λ*_3 _= 0.5, *λ*_4 _= 0.1, *λ*_5 _= 12, *μ_A _*= 10, *μ_B _*= 0, *δ_A _*= 0.01, *δ_B _*= 0.1. Using the exact formula (9a) from the text and the standard numerical PDE solver, I obtain the following figure (Figure 9).

Here the blue line shows the exact solution for *A*_0 _(*x*) of the corresponding linear system, the red line shows a numerical solution for *A *(*x*) of the full nonlinear system for large *t*. This figure gives a clear example that it is dangerous to make any conclusions about the long-term behavior of the nonlinear system using the exact solutions of the approximate linear system. From a mathematical standpoint it is only possible to state that the solutions of two systems are *ε*-close on the times of order *O*(1/*ε*) but nothing can be proved for *t *→ ∞. In general, the authors should either not consider the exact solution of the approximate linear system or specify the parameter domain in which the linear approximation works.

Response: *We thank the reviewer for drawing our attention to this point. As is stated in the section on the analysis of the small λ*_1 _*case, we need to assume that the other parameters are O*(1) *for analysis to be valid, and we have now reiterated this at the beginning of the appendix. (In passing, we remark that in some cases, the approximate, small-λ*_1 _*solution gives good agreement even when this assumption is violated; however, this cannot be expected in general.) We note that in the case presented by the reviewer, the ratio μ_A_/δ_A _(which appears in the equation for A) is O*(10^3^)*; hence the grouping λ*_1 _*μ_A_/δ_A _is not small, and we would not expect our analysis to be valid. However, we have now included an example of this type of behaviour (the new *Figure 7*) in the numerical results section, as it demonstrates the important point that there are situations where a small value of λ*_1 _*can nevertheless make a significant difference to the solution. For the cases where the other parameters are O*(1), *we have compared our analysis with the numerics for several cases. The agreement with the leading-order solution is generally reasonable, and improves with the addition of the first order correction term. However, since this does not really provide any new information to the reader, the relevant plots are omitted*.

#### Minor Comments

1. Page 2, second column. ". . . the drug must the. . . " should be ". . . the drug must be."

2. Page 3, second column. Why use indexes for the initial conditions?

3. Page 5, second column. ". . . exactly by (8). . . " should be ". . . exactly by (9)."

4. Page 6, second column. A missing number in ". . . in the expansion ()."

5. The first reference I checked has a typo: the paper by Zhang et al. was published in 2010, not in 2009. Please check all the other references.

Response: *We thank the reviewer for also pointing out a number of smaller errors, which we have corrected according to their suggestions, except for retaining the subscript i for initial conditions, where we think the intended meaning is clear*.

### Reviewer's Report 2

Dr William Hlavacek

#### Main Comments

The authors present steady-state results, including analytical results, for a model consisting of three partial differential equations for a system in which there is a reversible bimolecular association reaction (A+B = C) and one-dimensional diffusion of the chemical species A, B and C. The analytical results are approximate and obtained through a perturbative approach. The analytical expressions given by the authors are fairly complicated, and it is unclear that these expressions provide more insight than purely numerical results. The model is simple, but the authors explain that models even simpler in some respects have been considered in earlier related work and that their study is relevant for understanding penetration of the activated form of a prodrug into a solid tumor and for understanding growth factor action in tissues. The report is well written. The insights gained from analysis of the model seem somewhat obvious. For example, the authors conclude from their analysis that rapid clearance of the activated form of a prodrug will decrease its effectiveness. I'm not sure anyone would need a model to understand this point. Also, it is unclear that any of the insights gained from the analysis are actionable. For example, the authors suggest a piggy-backing strategy for increasing drug penetration but no concrete example is provided. Can the authors provide more details about potential proteins that could serve as carriers of drugs into solid tumors? In any case, the results are purely theoretical. There is no demonstration that the results are useful in practice. There is only limited discussion of parameter estimates.

Response: We thank the reviewer for their report. They note that the fact that e.g. increasing the rate of active drug clearance tends to reduce its concentration in the tissue is intuitively clear. We agree, but would point out that there are phenomena which can be observed in the model which may not be so clear on an intuitive level. For example, the fact that the maximum drug concentration may occur at a point intermediate between the centre and the edge of the tissue. If such an observation was made experimentally, we do not believe it would necessarily be intuitively obvious that it could be produced by the simple mechanism outlined here. Of course, many other processes may produce such concentration profiles, and each case must be considered individually. However, we believe in general it would be sensible to consider the possibility of a scenario of the type we have suggested. We also agree that the analytical expressions (which are given for the sake of completeness) are somewhat complicated, and in are best illustrated by plots as for the numerical results. However, we believe they can give useful information about the dependence of the solution on the various parameters. For example, we see there is a maximum theoretically attainable active drug concentration (A* + D_C _C*/D_A _in dimensional terms). This is potentially useful practically, since if this level is below that at which the drug is effective, alternative strategies for drug delivery will need to be investigated. Similarly, our model suggests a framework by which simple in vitro *experiments might be used to characterise which are the most promising drug candidates for development by measuring parameters such as their diffusion coefficients and reaction rates: e.g. those with the smallest value of λ*_1 _*or λ*_3 _*or largest λ*_2 _*appear more likely to give rise to effective treatments. We note the recent study announcing negative results in a trial of TPZ in treating advanced squamous cell carcinoma of the head and neck, and have included a reference to this paper. However, the argument we wished to make in our paper was a general one: that considering merely the concentration of the prodrug (C in our model) could be misleading, since the concentration profile of the active drug (A) in the tissue could be very different. This applies to any drug which is delivered in the manner considered here. Owing to lack of experimental data, we cannot make any definitive statements about the effectiveness of TPZ, and the reference to it was merely offered as a possible example. We have re-written this paragraph of the 'Background' section to try to make our intention clearer*.

### Reviewer's Report 3

Dr James Faeder

#### Main Comments

This paper presents a simple mathematical model to describe the spatial distribution of drug in tissue where the drug may interact with another molecule (e.g. protein) to form a complex that undergoes degradation at a different rate from the drug alone, producing back again the active drug and eliminating the binding partner. The appropriate partial differential equations to describe this system in the continuum limit are derived and solved exactly or approximately in limiting cases, and these solutions are supplemented by the use of numerical simulation on the full model. The main findings are that under appropriate conditions, e.g., when the degradation of active drug is relatively slow, the breakdown of the complex is fast, and the rate of complex formation is slow relative to the diffusion constant of the drug, the concentration profile of the drug in tissue reaches a maximum at the point farthest away from the boundaries (under the assumption of fixed concentrations on the boundaries and a symmetric, 1D tissue). Such a profile suggests a potential novel mechanism for the activity of a drug that must be targeted selectively to a tissue by utilizing enhanced tissue-specific degradation of a complex between the drug and another molecule or protein. Alternatively, some other tissue-specific factor, such as hypoxia in the case of a tumor, may be used for rate-enhancement of the activating process. I found this paper to be very clearly written both at a general and technical level. The findings are also clearly significant, both for their potential therapeutic implications and for modelers interested in applying this model or extensions of it to model other systems. I have major criticisms of the paper. - At the bottom of 2nd column on p. 3., I found the sentence "In this example, b does not play an active role. . . " to be confusing. I think it would help to explain the application of the model to the prodrug example a bit more clearly. Specifically, how is b modeled in that case?

-There is a typo in Eq. 8c, which is missing '=0' before the period.

Response: *We thank the reviewer for their positive report. We have addressed the minor matters raised in the review*.

## A Details of the solutions for *A*_1_, *B*_1 _and *C*_1 _given in equation (10)

For the sake of completeness, we give here the details of the solutions for the first-order correction terms (note that we assume that the other parameters, apart from *λ*_1 _are *O*(1)). Substituting the expansions (7) into the governing equations (4), at *O*(*λ*_1_), we find

$$\begin{array}{c} \frac{\partial^{2}A_{1}}{\partial x^{2}}-\frac{\mu_{A}}{\delta_{A}}A_{0}B_{0}+\frac{\lambda_{2}\mu_{A}}{\delta_{A}}C_{1}-\frac{\lambda_{3}}{\delta_{A}}A_{1}=0,\\ \frac{\partial^{2}B_{1}}{\partial x^{2}}-\frac{\mu_{B}}{\delta_{B}}A_{0}B_{0}-\frac{\lambda_{4}}{\delta_{B}}B_{1}+\frac{\lambda_{5}}{\delta_{B}}C_{1}=0,\\ \frac{\partial^{2}C_{1}}{\partial x^{2}}+A_{0}B_{0}-\lambda_{2}C_{1}. \end{array}$$

The above are to be solved subject to the boundary conditions

$$\begin{array}{c} \frac{\partial A_{1}}{\partial x}=\frac{\partial B_{1}}{\partial x}=\frac{\partial C_{1}}{\partial x}=0\;\text{at }x=0,\\ A_{1}=B_{1}=C_{1}=0\;\text{at }x=1. \end{array}$$

The general form of the solution to the above is given by (10), whilst the details of the coefficients are provided below. The $A_{j}$ and $A^{*}$ are

Aj=-μACjδAθj2-λ3δAfori=1,…,6,A7=-4λ2μAC7δA4λ2-λ3δA,A8=-μAλ2C*δAλ2-λ3δA,A*=-1coshλ3δA∑j=16Aj coshθj+A7 cosh2λ2+A8 cosh(λ2x)

where the *θ_j _*are given by equation (11), and the coefficients $C_{j}$ and $C^{*}$ are given below.

The coefficients in the expression for *B*_1 _are given by

B1=1δBθ12-λ4δBμBβ1β32-λ5C1,B2=1δBθ22-λ4δBμBβ1β32-λ5C2,B3=1δBθ32-λ4δBμBβ1β42-λ5C3,B4=1δBθ42-λ4δBμBβ1β42-λ5C4,B5=1δBθ52-λ4δBμBβ2β32-λ5C5,B6=1δBθ62-λ4δBμBβ2β32-λ5C6,B7=1δB4λ2-λ4δBμBβ2β42-λ5C7,B8=1λ4-μBβ2β42+C8,B9=-λ5C*δBλ2-λ4δB,B*=-1coshλ3δA∑j=16Bj coshθj+B7 cosh2λ2+B8+B9 cosh(λ2x),

where we have introduced

$$\begin{array}{c} \beta_{1}=\frac{1}{\cosh\sqrt{\frac{\lambda_{3}}{\delta_{A}}}}\left(1+\frac{\lambda_{2}\mu_{A}}{\delta_{A}\lambda_{2}-\lambda_{3}}\right),\\ \beta_{2}=-\frac{1}{\cosh\sqrt{\lambda_{2}}}\left(\frac{\lambda_{2}\mu_{A}}{\delta_{A}\lambda_{2}-\lambda_{3}}\right),\\ \beta_{3}=\frac{1}{\cosh\sqrt{\frac{\lambda_{4}}{\delta_{B}}}}\left(1+\frac{\lambda_{5}}{\delta_{B}\lambda_{2}-\lambda_{4}}\right),\\ \beta_{4}=-\frac{1}{\cosh\sqrt{\lambda_{2}}}\left(\frac{\lambda 5}{\delta_{B}\lambda_{2}-\lambda_{4}}\right). \end{array}$$

Finally, the coefficients in the solution for *C*_1 _are

C1=-β1β32(θ12-λ2),C2=-β1β32(θ22-λ2),C3=-β1β42(θ32-λ2),C4=-β1β42(θ42-λ2),C5=-β2β32(θ52-λ2),C6=-β2β32(θ62-λ2),C7=-β2β46λ2,C8=β2β42λ2,C*=-1coshλ2∑j=16Cj coshθj+C7 cosh2λ2+C8.

We note, as is clear from the above, that the solution (10) is only valid provided $\theta_{i}^{2}\neq \frac{\lambda_{3}}{\delta_{A}}$*etc*.; however, the solutions in the special cases where this doesn not apply can be found from (10) by straightforward application of L'Hopital's rule.
